# Optimizing MRI sequences and apparent diffusion coefficient parameters for small pancreatic ductal adenocarcinoma detection

**DOI:** 10.1007/s10689-025-00447-x

**Published:** 2025-03-07

**Authors:** Naoko Mori

**Affiliations:** Department of Radiology, Akita University Graduate School of Medicine, 1-1-1 Hondo, Akita, 010-8543 Japan

**Keywords:** Pancreatic cancer, MRI, Diffusion-weighted imaging, Apparent diffusion coefficient, T1-weighted imaging

To the editor

I read with interest the article by Boekestijn et al., “Screening for pancreatic cancer in high-risk individuals using MRI: optimization of scan techniques to detect small lesions” [1]. They discuss the optimization of MRI techniques for early detection of pancreatic ductal carcinoma (PDAC) in situ in high-risk individuals. Their study emphasizes the importance of detecting small PDAC (less than 10 mm) to significantly improve survival rates. The review highlights advances in MRI hardware and software, the refinement of dedicated imaging protocols, and the potential role of artificial intelligence in improving the quality of screening programs for PDAC. I agree with the study of Boekestijn et al., which shows the role of different MRI sequences in identifying small lesions and emphasizes their contributions to improved screening outcomes. I would like to add a discussion on the combination of MRI sequences that can effectively detect small PDAC. Additionally, I would like to emphasize the usefulness of apparent diffusion coefficient (ADC) parameters in diffusion-weighted imaging (DWI) for differentiating between PDAC and autoimmune pancreatitis.

We conducted an approach using T1-weighted images (T1WI), T2-weighted images (T2WI), magnetic resonance cholangiopancreatography (MRCP), and DWI to detect small PDAC and examined which combination of sequences was most effective in detecting small PDAC [2]. As a result, we reported that using an ‘OR’ combination approach, where findings in T1WI, DWI, or MRCP individually suggest small PDAC, resulted in the highest sensitivity. The “OR combination” means that if findings are observed in any of the T1WI, DWI, or MRCP, the likelihood of small PDAC is considered high. On the other hand, findings from T2WI, such as the presence of a mass or dilatation of the main pancreatic duct, did not contribute to improvements in sensitivity or specificity. It is possible that the observations from T2WI, such as the presence of a mass or dilatation of the main pancreatic duct, may already be captured by T1WI, DWI, or MRCP. In screening for small PDAC, it is essential to use an imaging protocol that is both time-efficient and non-invasive. A minimal sequence protocol may be required. Further prospective studies are needed to investigate the combination of sequences that would be useful in screening for small PDAC.

Additionally, their review article states that the utility of DWI in distinguishing between small PDAC and mass-forming autoimmune pancreatitis is still unclear. They mention that even with the use of the apparent diffusion coefficient (ADC), it remains challenging to differentiate between the two groups. We conducted a histogram analysis of ADC values and reported that the maximum ADC value is optimal for distinguishing small PDAC from mass-forming autoimmune pancreatitis [3]. In the future, it will be necessary to optimize the ADC parameters to confirm the usefulness of DWI in the differentiation of small PDAC.

Once again, I appreciate the work by Boekestijn et al. in improving MRI technology and scan quality for early pancreatic cancer detection, contributing to better prognosis in screening programs.

## Data Availability

No datasets were generated or analysed during the current study.
